# Millet and meals: the role and significance of *Panicum miliaceum* in culinary contexts at Bruszczewo, Poland

**DOI:** 10.1007/s12520-024-02095-1

**Published:** 2024-12-04

**Authors:** Edward A. Standall, Oliver E. Craig, Jutta Kneisel, Johannes Müller, Wiebke Kirleis, Janusz Czebreszuk, Carl Heron

**Affiliations:** 1 Department of Scientific Research, British Museum, London, WC1B 3DG UK; 2 BioArCh, Department of Archaeology, University of York, York, YO10 5DD UK; 3 Institute of Pre- and Protohistory, Kiel University (Christian-Albrechts-Universität zu Kiel), 24118 Kiel, Germany; 4 Faculty of Archaeology, Adam Mickiewicz University, Poznań, 61-614 Poland; 5 Nara National Research Institute for Cultural Properties, Nara, 630-8577 Japan

**Keywords:** Bronze Age, Miliacin, Organic Residue Analysis (ORA), Ceramic, Foodcrust, GC-MS

## Abstract

The translocation of broomcorn millet (*Panicum miliaceum*) is an enticing subject of archaeological investigation. While the species was rapidly dispersed across Eurasia during prehistory, its adoption appears to have been inconsistent, with evidence for its sudden and gradual adoption, as well as its rejection, observed within and between contemporary communities and cultures. The translocation of *P. miliaceum* may be better understood through the identification of its role and significance as a foodstuff among past populations. This study employs molecular investigation and bulk and compound-specific isotope analysis to study organic residues produced during culinary activities at Bruszczewo, Poland, during the Early Bronze Age (EBA) and Late Bronze Age / Early Iron Age (LBA/EIA). The analysis of ceramic-absorbed and charred ‘foodcrust’ residues enabled a comprehensive examination of vessel use-life and the production of individual meals. This study strongly suggests that *P. miliaceum* was not present at Bruszczewo during the EBA, conforming to its established translocation chronology, while highlighting its extensive use during the LBA/EIA. The cereal was incorporated into a variety of culinary activities, constituting both a minor and major ingredient of many meals, while being absent in others. Finally, the observation of apparently unrestricted use of *P. miliaceum* at Bruszczewo provided further insight into the role and significance of this cereal relative to contemporary LBA/EIA evidence for its use across the region.

**Supplementary Information:**

The online version contains supplementary material available at 10.1007/s12520-024-02095-1.

## Introduction

The translocation of broomcorn millet (*Panicum miliaceum*) across northern Eurasia has been the subject of increasing archaeological investigation in recent years. Molecular (Heron et al. 2016; Isaksson and Nilsson 2018; Rageot et al. 2019; Murakami et al. 2022), isotopic (Hermes et al. 2019; Pospieszny et al. 2021), and genetic analyses (Hunt et al. 2011, 2014, 2018), in addition to direct radiocarbon dating (Motuzaitė Matuzevičiūtė et al. 2013; Filipović et al. 2020; Dal Corso et al. 2022; Alonso and Pérez-Jordà 2023; Motuzaitė Matuzevičiūtė and Laužikas 2023), have complemented archaeobotanical investigations and developed our understanding of the introduction, adoption, and significance of this cereal across Eurasia during prehistory (Kirleis et al. 2022).

Archaeobotanical and isotopic investigations demonstrate inter- and intra-cultural variation in the adoption of *P. miliaceum* across Europe (Filipović et al. 2022), with concurrent introduction and mass adoption, gradual adoption after introduction, and non-adoption identified at contemporary sites in Greece (Valamoti 2016), Italy (Perego 2015; Tafuri et al. 2018), Poland (Kapcia and Mueller-Bieniek 2019; Pospieszny et al. 2021), and Germany (Effenberger 2018). The low number of direct radiocarbon dates across Europe limits our ability to assess the rate and, as such, the nature of adoption among different cultures and communities, although this is being addressed in large-scale dating projects (Filipović et al. 2020). Nonetheless, sub-regional datasets indicate varied attitudes and approaches towards *P. miliaceum* that are perhaps unlikely to be exclusively explained by either ecological opportunism or economic principles (Jones et al. 2011; Lightfoot et al. 2013; Filipović et al. 2022). Indeed, evidence suggests that cultural factors and localised decision-making significantly influenced the adoption of *P. miliaceum* across Europe. However, one must also consider that economic and cultural significance are not mutually exclusive, and that the status of the cereal may have changed over time (Boivin et al. 2012).

A novel method of investigating factors that influenced the translocation of *P. miliaceum* is organic residue analysis (ORA). Individual, social, and cultural identity may be displayed through the selection and combination of ingredients and use of different cooking methods and equipment during the production of meals (Hastorf 2017). As such, the analysis of organic residues, extracted from culinary equipment and the remnants of past meals, enables the identification and understanding of *P. miliaceum* use as a foodstuff and further inference of the role and significance of the cereal among past populations.

Heron et al. (2016) first proposed ORA to identify the use of *P. miliaceum* in culinary activities, developing on an approach previously applied in sedimentological studies (Jacob et al. 2008). The investigation of *P. miliaceum*, via ORA, is possible due to the production and concentration of a pentacyclic triterpene methyl ether (PTME), miliacin (olean-18-en-3β-ol methyl ether), in its caryopses. While this compound is not exclusively produced by *P. miliaceum*, it may be considered as a species-specific biomarker depending on geographical, chronological, and culinary context (Jacob et al. 2008; Bossard et al. 2013; Standall et al. 2022). A secondary means of investigation, albeit one that is less specific and sensitive than the biomarker approach, is carbon isotope analysis. As a C_4_ plant, *P. miliaceum* is isotopically distinct from most other (C_3_) plants, which are the dominant carbon sources in northern Eurasia (Lightfoot et al. 2013), although it is indistinguishable from marine resources.

Heron et al. (2016) identified *P. miliaceum* processing at Bruszczewo, Poland, in their analysis of 61 Early Bronze Age (EBA) and Late Bronze Age / Early Iron Age (LBA/EIA) charred crusts (foodcrusts) analysed by elemental analysis - isotope ratio mass spectrometry (EA-IRMS), gas chromatography - mass spectrometry (GC-MS), and gas chromatography - combustion - isotope ratio mass spectrometry (GC-C-IRMS). While substantial ^13^C enrichment was observed in over half of the LBA/EIA samples analysed by EA-IRMS, and in the only LBA/EIA sample analysed by GC-C-IRMS, ^13^C enrichment was not observed in any EBA sample. This corresponded to the presence and absence of miliacin in one LBA/EIA and one EBA charred crust respectively and is consistent with the translocation chronology of *P. miliaceum* throughout Europe, as determined by direct radiocarbon dating (Motuzaitė Matuzevičiūtė et al. 2013; Filipović et al. 2020). However, as miliacin was only identified in one charred crust, wider understanding of the role and significance of *P. miliaceum* at Bruszczewo is limited. Consequently, further research is necessary to determine the extent to which the cereal was used, how it was incorporated into culinary activities, and how its use related to material culture.

The aim of this paper is to achieve a more complete understanding of culinary activities at Bruszczewo. This study develops on previous analyses by increasing the number of charred crusts analysed by EA-IRMS, GC-MS, and GC-C-IRMS, in addition to applying GC-MS and GC-C-IRMS to 42 ceramic-absorbed residues. Data from this study and Heron et al. (2016) are used in the interpretation of culinary activities at Bruszczewo. The analysis of charred crusts and ceramic-absorbed residues, in several instances from the same vessel, enabled a nuanced understanding of individual meals and long-term culinary processes. This paper also considers the reliability of criteria used in the identification of *P. miliaceum* in archaeological organic residues. This study is driven by the following research questions (Table 1).

**Table 1:** Research aims and objectives

Question	How can this be established?
Is the apparent absence of *P. miliaceum* in the EBA supported by the analysis of a larger and more diverse sample set?	By demonstrating an absence of miliacin and both bulk and compound-specific ^13^C enrichment, indicative of C_4_ plant influence, in EBA samples.
How prevalent was *P. miliaceum* processing in the LBA/EIA?	By assessing the frequency and abundance of miliacin, and the frequency and extent of significant bulk and compound-specific ^13^C enrichment, in LBA/EIA samples.
How was *P. miliaceum* prepared, consumed, and incorporated into culinary culture?	By comparing the molecular and isotopic composition of charred crust and ceramic residues and by incorporating ceramic typology into the interpretation of ORA data.

## *P. miliaceum* in a regional context

Direct radiocarbon dates have been obtained for six *P. miliaceum* caryopses recovered from early archaeological contexts in Poland (Filipović et al. 2020). The earliest date is associated with MBA activity, ca. 1300 cal BC, at Lipnik, southeast Poland (Kapcia and Mueller-Bieniek 2019). However, bulk isotope analysis of human remains indicates the consumption of ^13^C-enriched foodstuffs in southeast Poland from the mid-15th c. BC (Pospieszny et al. 2021). This corresponds to substantial archaeobotanical evidence for *P. miliaceum* in MBA contexts (Moskal-del Hoyo et al. 2015), indicating that the cereal was well established in this region earlier than is suggested by current radiocarbon dates. Isotopic data also indicates selective consumption of ^13^C enriched foodstuffs within and between communities, perhaps suggesting localised attitudes towards the cereal (Pospieszny et al. 2021). However, it is not presently possible to confidently state whether humans regularly consumed *P. miliaceum* directly or if ^13^C enrichment is derived from animals that consumed the cereal.

The earliest direct radiocarbon date for *P. miliaceum* in central Poland is from Lutomiersk-Koziówki, ca. 1000 cal BC, corresponding to the LBA (Mueller-Bieniek et al. 2015). Interestingly, there is no evidence for the consumption of ^13^C enriched foodstuffs in central Poland during the entire Bronze Age (Pokutta and Howcroft 2015; Pospieszny et al. 2021), despite substantial archaeobotanical evidence for its cultivation. However, while isotopic data may reflect a difference in the nature and extent of *P. miliaceum* use in this region, both methodological and practical limitations of this technique should be considered before reaching a conclusion (Lee-Thorp 2008). For instance, the prevalence of cremation burials increases towards the end of the Bronze Age, limiting material available for analysis and potentially introducing bias into isotopic datasets (Pospieszny et al. 2021).

## Site background

**Fig. 1:**
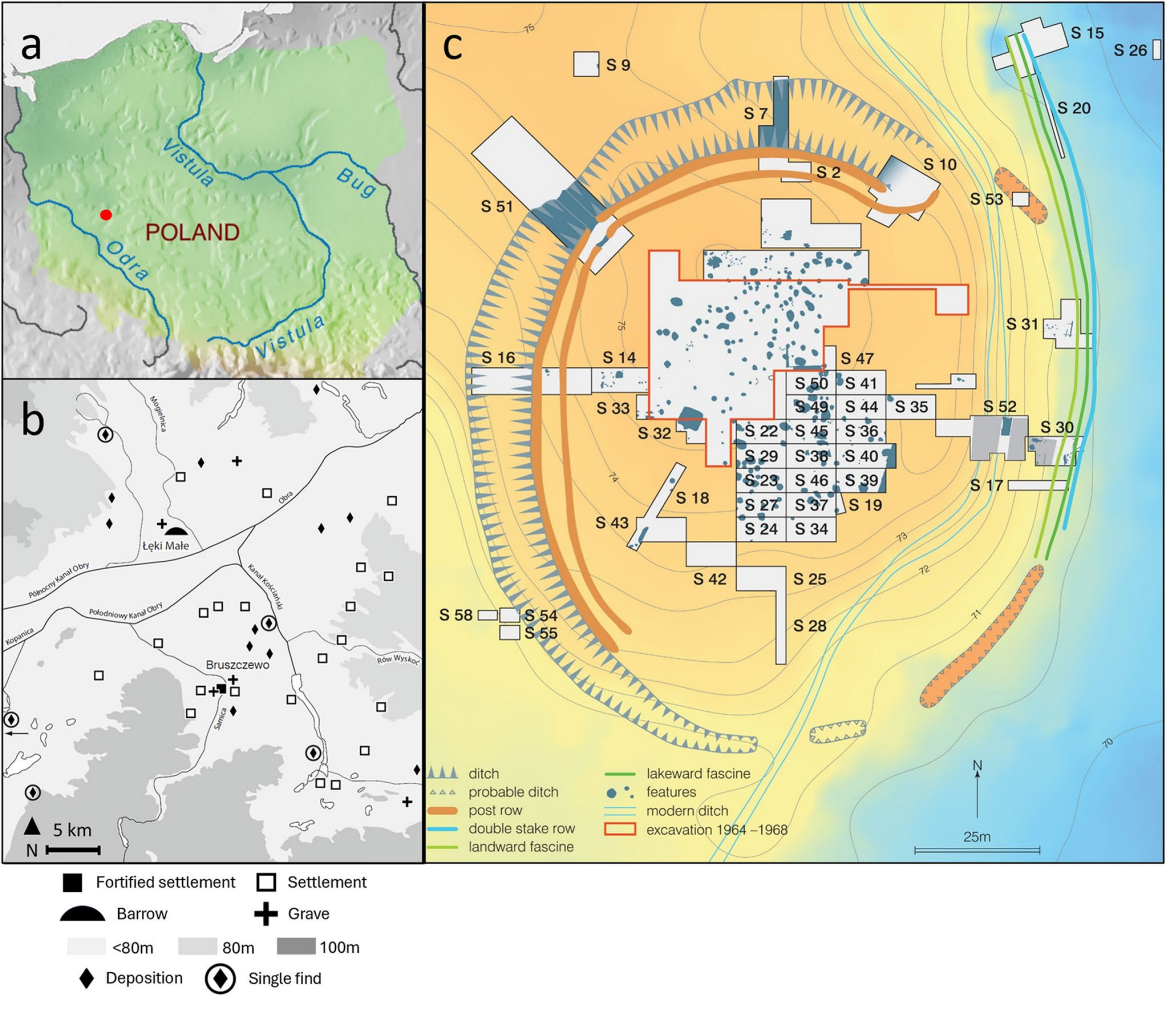
The location of Bruszczewo within Poland (**a**), distribution of EBA activity recorded in close proximity to Bruszczewo (**b**, Müller and Kneisel 2010. 761), and plan of the EBA settlement (**c**, Müller and Kneisel 2010, 753)

The archaeological site of Bruszczewo 5 is situated on an elevated spur of land in the Samic River Valley of Kościan County, Poland (52°00′ 47″ N, 16°35′ 07″ E, Heron et al. 2016; Fig. 1). Prehistoric activity is observed in two distinct periods, including EBA Únĕtice Culture activity between 2000 − 1500 cal BC and LBA/EIA Lusatian Culture activity around 960 cal BC (Czebreszuk et al. 2015).

The EBA settlement (Fig. 1) occupied a prominent location that was extensively developed and fortified and was situated near several rich burials (Łęki Małe and Przysieka Polska), indicating that Bruszczewo was a focal point in the Kościan region. The recovery of amber artifacts demonstrates a role in long distance exchange networks and metal artifacts indicate the presence of an elite metalworking community (Müller and Kneisel 2010; Czebreszuk et al. 2015; Jaeger and Stróżyk 2015). Although isolated *P. miliaceum* caryopses have been recovered from EBA contexts, they are presumed to be intrusive from overlying LBA/EIA contexts, as has been extensively demonstrated elsewhere (Motuzaitė Matuzevičiūtė et al. 2013; Filipović et al. 2020). Of less certain origin are several ceramic sherds that possess hundreds of supposed *P. miliaceum* caryopsis impressions (Kroll 2010; see An et al. 2019), exclusively on their interior surface, as they were recovered from an intermediary layer between EBA and LBA/EIA contexts. Given the apparent significance of Bruszczewo during the EBA, its near contemporary occupation with the apparent introduction of *P. miliaceum* to Poland, and potential evidence for the cereal in EBA contexts, one must consider whether the cereal was introduced to the site at an earlier date than the regional chronology of its introduction suggests.

After several centuries of disuse, the LBA/EIA site was established atop the derelict EBA settlement. Lusatian Culture activity is evidenced by a limited number of archaeological features, preventing confident interpretation of site function, with both habitation and funerary activities proposed as potential uses (Ignaczak 2015). The ceramic assemblage is atypical of Lusatian Culture settlements, with bowls, cups, and plates the most common forms, pots and vases being rare, and jugs notably absent despite their ubiquity at most Lusatian Culture settlements. This distribution of ceramic types is consistent with contemporary cemeteries, e.g., Kietrz and Laski, and corresponds to an increase in the prevalence of drinking and feasting at ceremonial and funerary sites across Europe during the 13th -12th c. BC (Ignaczak 2015). The frequency and abundance of *P. miliaceum* caryopses recovered is substantially greater in this period than the EBA, yet they are concentrated in a limited number of features, wherein they dominate, and are a minor component of the overall assemblage relative to barley (Kroll 2010).

## Sampling strategy

Sampling was focussed on well-defined EBA and LBA/EIA material. Ceramic sampling was prioritised, although charred crusts were obtained where possible, enabling both direct and indirect comparison of molecular and isotopic data obtained in this study and by Heron et al. (2016). Samples were taken from the internal surface of predominantly rim and body sherds derived from various vessel types. The sherds were obtained from three trenches located in the eastern peat zone (S30, S31, and S52, Fig. 1) that demonstrated exceptional preservation of organic materials. In total, 62 samples were obtained, including 42 ceramic samples from 20 EBA and 22 LBA/EIA sherds, 19 charred crusts from 9 EBA and 10 LBA/EIA sherds, and one ‘charred crust’ obtained from an amorphous charred mass recovered from the base of an EBA sherd. Laboratory codes starting with Br 0## and Br 1## were given to EBA and LBA/EIA samples respectively.

Methods used in the preparation, extraction, analysis, and interpretation of samples are detailed in Online Resource 1.

## Results and discussion

### EA-IRMS analysis

**Fig. 2:**
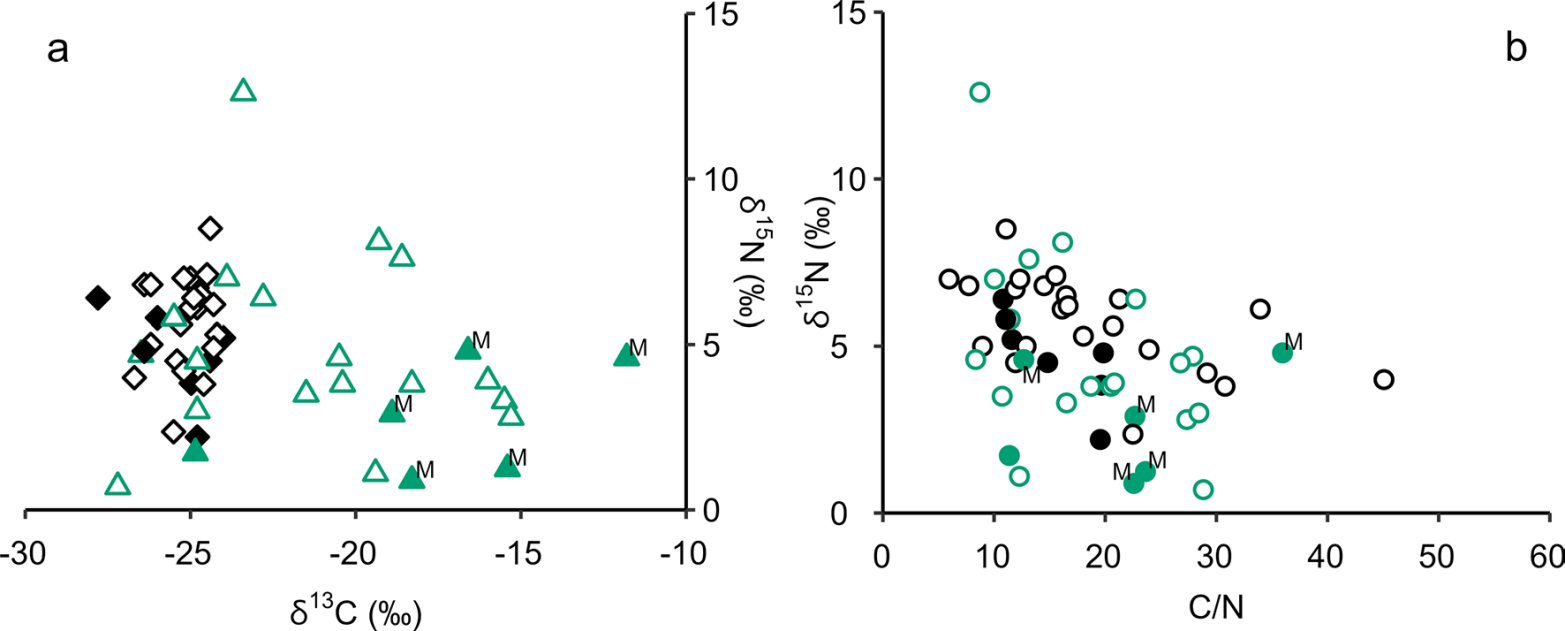
Plots of δ^15^N versus δ^13^C (**a**) and δ^15^N versus atomic C/N (**b**) values of EBA (**black**) and LBA/EIA (**green**) charred crusts. Filled points represent samples analysed by GC-MS. **M** = LBA/EIA charred crusts that contain miliacin. Data is presented in Online Resource 2

Fourteen charred crusts were subjected to EA-IRMS, with results obtained on five EBA and three LBA/EIA samples. Six samples were rejected due to insufficient carbon content (< 10% by weight), in-line with Heron et al. (2016). Data from this study and Heron et al. (2016) are combined in Fig. 2; Table 2. The new EA-IRMS data is consistent with previous results. A significant difference is observed between EBA and LBA/EIA δ^13^C (unpaired t-test, t = 6.1319, df = 51, *p* < .0001) and δ^15^N (unpaired t-test, t = 3.1051, df = 50, *p* = .0031) values, provided that the δ^15^N value of sample F7061ID4765 is excluded as an outlier in the LBA/EIA dataset (Z-score = 3.18).

**Table 2:** Summary of bulk δ^13^C, δ^15^N, and atomic C/N values of charred crusts from Bruszczewo. Data is presented in Online Resource 2

	EBA			LBA/EIA		
	δ^13^C (‰)	δ^15^N (‰)	C/N	δ^13^C (‰)	δ^15^N (‰)	C/N
*n*	29			25		
Mean (1 s.d.)	-25.2 ± 0.9	5.5 ± 1.4	17.8 ± 8.4	-20.4 ± 4.0	4.4 ± 2.5	19.1 ± 7.5
Range	-24.0 to -27.8	2.2 to 8.5	6.0 to 45.1	-11.8 to -27.2	0.9 to 12.6	8.3 to 35.9

The bulk δ^13^C values of EBA samples are within the range expected for C_3_ carbon sources, with generally low δ^15^N and high C/N values indicating the processing of primarily plant and terrestrial animal products, corresponding to archaeobotanical and zooarchaeological evidence from the site (Kroll 2010; Müller and Kneisel 2010; Makowiecki 2015). There is no evidence to suggest that ^13^C-enriched products contributed to the formation of EBA charred crusts. However, C_4_ plant products may not contribute a significant quantity of ^13^C to charred crusts when processed for short time periods and in unmodified states (Hart et al. 2007, 2009). Therefore, additional evidence is necessary to discount *P. miliaceum* processing in this period.

The bulk δ^13^C values of LBA/EIA samples are within the range of both C_3_ and C_4_ carbon sources, with half of those analysed demonstrating substantial ^13^C enrichment (δ^13^C > -22‰). The δ^13^C, δ^15^N, and C/N values obtained indicate the processing of plant and low trophic level animal products, in varying proportions, suggesting the production of varied meals. Sample F5017ID4992 may demonstrate exclusive processing of ^13^C-enriched products, as it produced a δ^13^C value (-11.8‰) close to that of an archaeological *P. miliaceum* caryopsis (-11.2‰, Motuzaitė Matuzevičiūtė et al. 2013). However, the source of ^13^C enrichment cannot be confirmed from this data, with the processing of *P. miliaceum* caryopses and ^13^C-enriched animal products feasible. The processing of marine resources is improbable given the low δ^15^N and high C/N values obtained from these samples, the distance of Bruszczewo from the coast, and the absence of evidence for these resources in the zooarchaeological record.

### GC-MS analysis

Molecular analysis was undertaken on 42 ceramic samples (20 EBA and 22 LBA/EIA) and 20 charred crusts (10 EBA and 10 LBA/EIA). All ceramic samples and all but one charred crust (B2) produced yields > 5 µg g^− 1^. A summary of the yields, relevant contents and isotopic composition of these samples is presented in Online Resource 2.

**Fig. 3:**
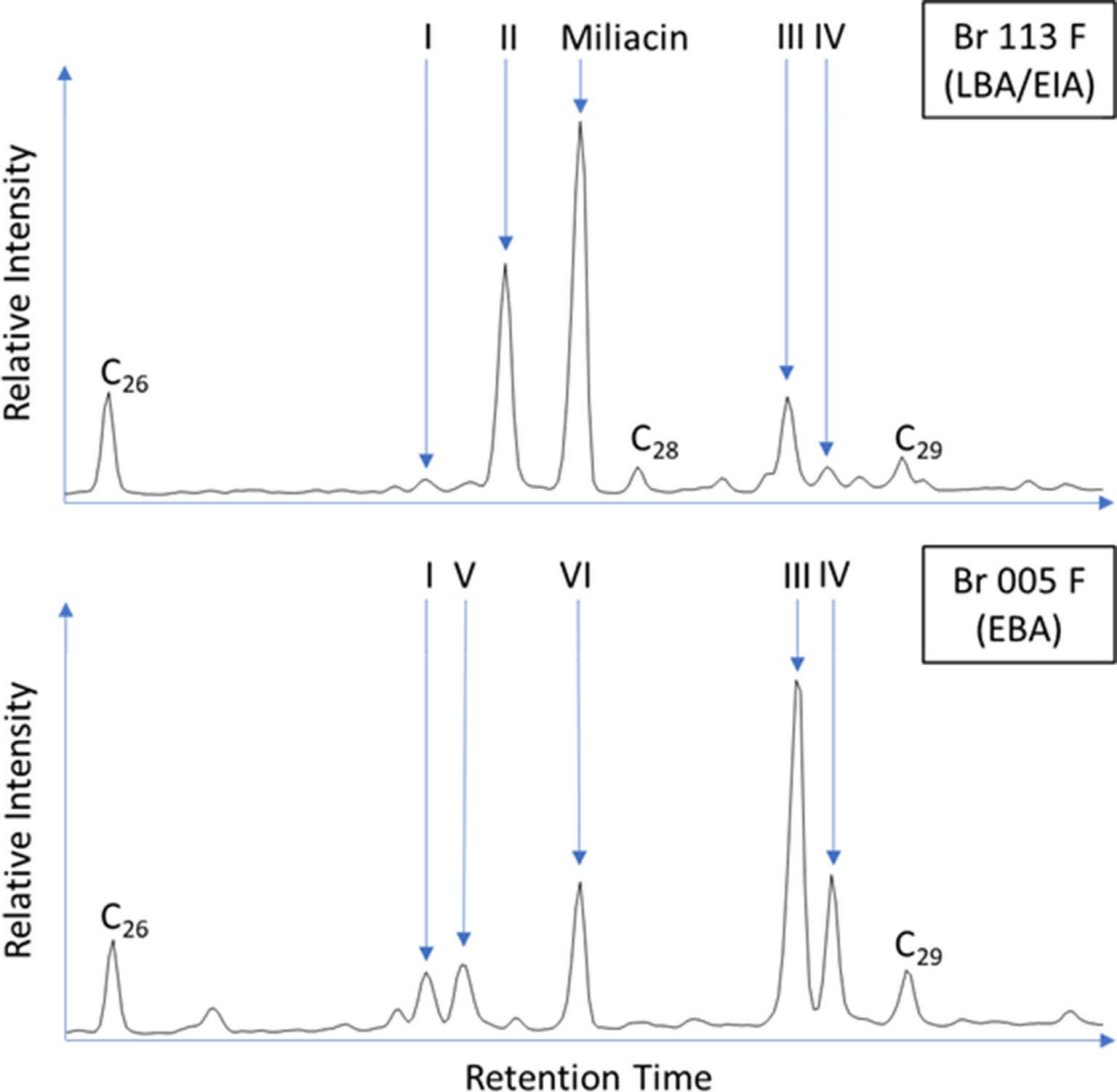
Partial TIC of Br 113 F and Br 005 F highlighting abundant long chain saturated fatty acids (**C**_**XX**_) and compounds indicative of plant product processing in foodcrusts. **I** Campesterol, **II** δ-Amyrin ME, Miliacin, **III** Sitosterol, **IV** Stigmastanol, **V** Ergostanol, **VI** Sitosterol ME

**Fig. 4:**
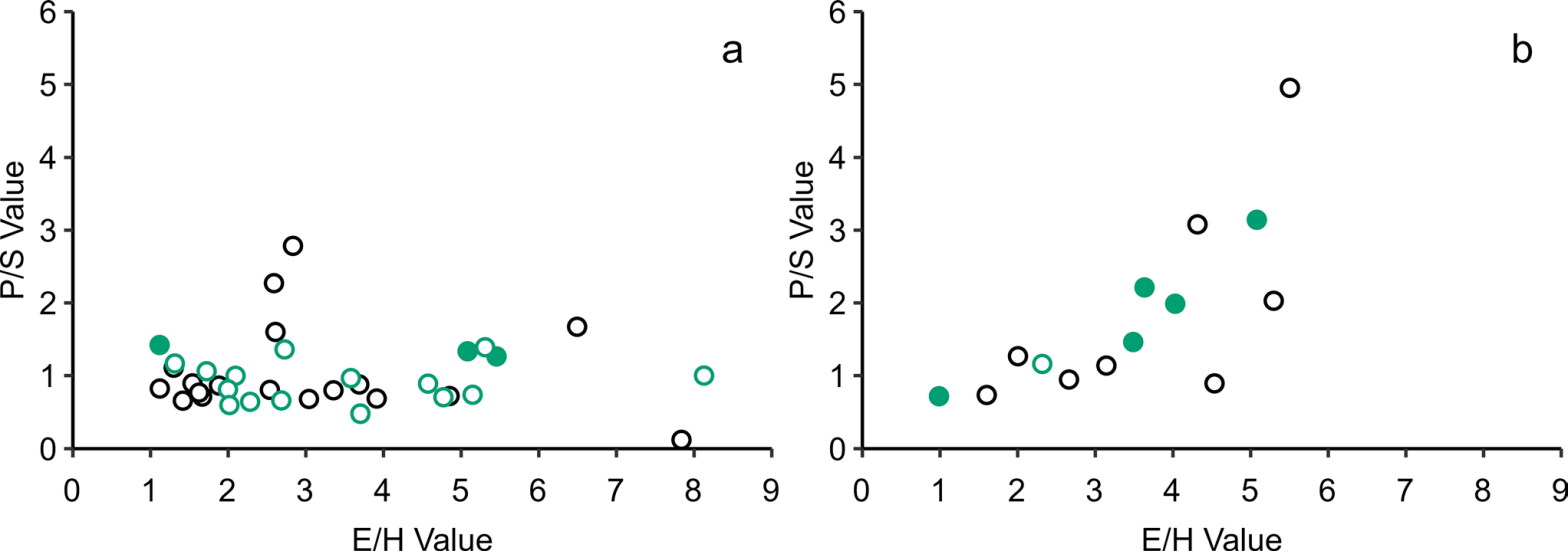
Plots of palmitic/stearic (P/S) acid ratios versus C_18_ APAA E/H isomer values from EBA (**black**) and LBA/EIA (**green**) ceramic-absorbed (**a**) and charred crust (**b**) residues. Filled circles contain miliacin. Data is presented in Online Resource 2

Ceramic-absorbed residues comprised a broad range of saturated (C_9:0_ - C_34:0_), unsaturated (C_16:1_ - C_24:1_ and C_18:2_) and branched chain (C_12_ - C_18_) fatty acids, with charred crusts comprising a slightly narrower range of compounds. Charred crust residues are generally dominated by palmitic acid (C_16:0_) and frequently contain phytosterol derivatives (Figs. 3 and 4), whereas ceramic-absorbed residues generally comprise a much lower abundance of palmitic acid (Fig. 4) and more frequently contain cholesterol derivatives. Pinaceae resin markers, retene, 7-oxodehydroabietic acid, and methyl dehydroabietic acid were identified at high abundances in the ceramic-absorbed residues of Br 013, 015, 104, and 106, demonstrating either the use of Pinaceae wood as fuel or the production/application of pitch (Simoneit et al. 2000; Brettell et al. 2015; Reber et al. 2019).

**Fig. 5:**
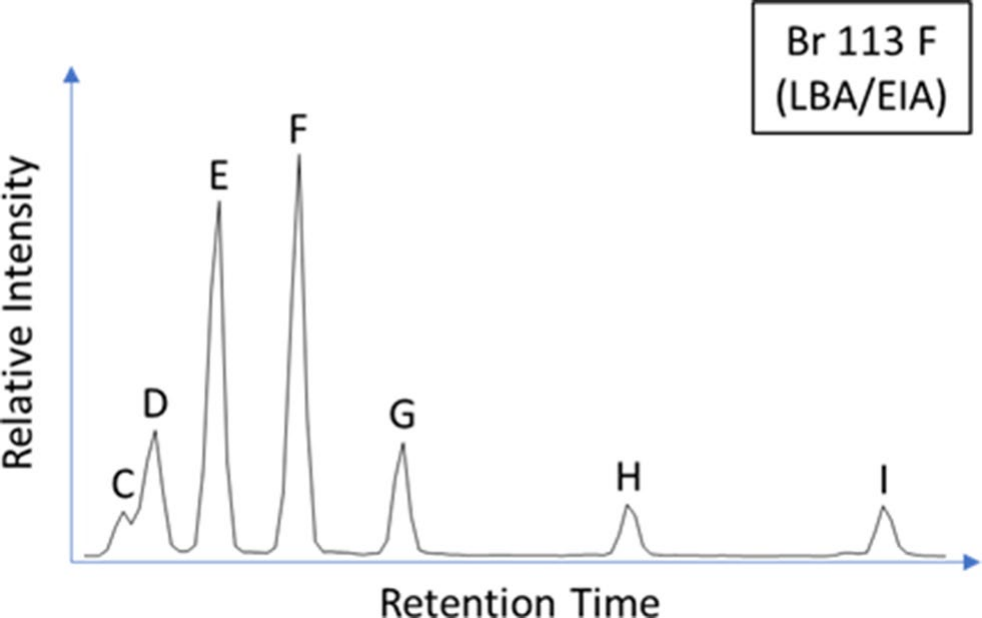
Partial EIC of *m/z* 290 highlighting a series of sequentially labelled C_18_ APAA isomers (Bondetti et al. 2021), present in Br 113 F. The ratio of E and H isomers (E/H) in this residue is 5.1

All extracts were subject to selected ion monitoring (SIM) to search for C_18_, C_20_, and C_22 _*ω*-(*o*-alkylphenyl)alkanoic acids (APAAs) and isoprenoid fatty acids 4,8,12-TMTD, phytanic acid and pristanic acid. Only C_18_ APAA isomers were observed, appearing more frequently and at higher abundances in charred crusts than ceramic-absorbed residues. Interestingly, not all charred crusts contained APAAs, indicating varied formation conditions, such as at high and low temperatures (Bondetti et al. 2021). The absence of C_20+_ APAAs and isoprenoid fatty acids further suggests that aquatic resources were not regularly processed in these vessels (Hansel et al. 2004; Copley et al. 2004). The relative abundances of E and H C_18_ APAA isomers, which are labelled according to their relative retention times (Fig. 5) but not structurally characterised, was assessed for each sample to help identify products that contributed to residue formation (Bondetti et al. 2021). Some EBA and LBA/EIA samples produced E/H values > 4 (Fig. 4), likely indicating the processing of either cereals, non-leafy vegetables, or fruits, although some non-ruminant adipose tissues may also produce these values (Bondetti et al. 2021).

While no relationship is observed between the E/H and P/S (palmitic/stearic acid) values of ceramic-absorbed residues (Fig. 4), a significant positive relationship exists between these values among charred crusts (r(12) = 0.74, *p* < .003). This relationship is strong and significant in LBA/EIA crusts (r(4) = 0.93, *p* < .007) and moderate but not significant in EBA crusts (r(6) = 0.69, *p* = .058). A difference in the significance of these relationships may reflect either the initial compositions of charred crusts or a degradation bias between palmitic and stearic acids, as APAAs are considered to degrade uniformly and retain their E/H values during deposition (Bondetti et al. 2021). Ceramic-absorbed residues likely represent an accumulation of lipids from multiple cooking events. Therefore, as specific conditions are required for APAA formation (Bondetti et al. 2021), it is possible that contradictory E/H and P/S values represent different processing methods, with variable APAA formation rates, applied to meals comprising ingredients with different P/S values.

Miliacin was not detected in any EBA sample. In contrast, miliacin was present in 16 of 22 ceramic-absorbed and 6 of 9 charred crust residues from LBA/EIA samples. Miliacin was present in all LBA/EIA charred crusts that exhibited bulk ^13^C enrichment and was absent in the one charred crust analysed that did not (Fig. 2). However, no relationship exists between bulk ^13^C enrichment and miliacin abundance, indicating a complex relationship between the two criteria. At the extremities of this dataset are samples B3, which contained the greatest quantity of miliacin (10.6% of the TLE) but produced the lowest bulk δ^13^C value (-18.9‰), and F5017ID4992, which contained only a trace of miliacin but produced the highest bulk δ^13^C value (-11.8‰). These results may indicate a source of ^13^C enrichment other than *P. miliaceum*, such as other C_4_ plants and ^13^C-enriched animal products. However, varied compositions of lipids, carbohydrates, and proteins, degradation biases between lipids, and complex mechanisms of miliacin accumulation may also explain these disparities.

Comparison of ceramic-absorbed and charred crust residues from Br 109 may demonstrate sequential processing of *P. miliaceum* and other foods, within the same vessel, as miliacin is present in the ceramic sample but not the charred crust. This would suggest that cooking vessels were multifunctional and used to process a variety of different meals, with *P. miliaceum* serving as one potential ingredient. This interpretation is supported by the disparity between E/H and P/S values of ceramic-absorbed and charred crust residues previously discussed, in addition to disparities in compound-specific δ^13^C values of ceramic-absorbed and charred crust residues, from the same vessels, discussed in the proceeding section.

### GC-C-IRMS analysis

Sufficient quantities of palmitic and stearic acids (> 30 ng per injection) were obtained from 31 ceramic-absorbed (13 EBA and 18 LBA/EIA) and 11 charred crust (6 EBA and 5 LBA/EIA) residues for compound-specific carbon isotope analysis. The mean, standard deviation, and range of these values are presented in Tables 3 and 4 for ceramics and charred crusts respectively.

**Table 3:** Summary of compound-specific δ^13^C values from EBA and LBA/EIA ceramic-absorbed residues. Data is presented in Online Resource 2

	EBA (*n* = 13)		LBA/EIA (*n* = 18)	
	δ^13^C_16:0_ ‰	δ^13^C_18:0_ ‰	δ^13^C_16:0_ ‰	δ^13^C_18:0_ ‰
Mean (1 s.d.)	-29.3 ± 1.3	-30.1 ± 1.5	-28.5 ± 1.3	-29.1 ± 1.2
Range	-26.1 to -31.1	-26.8 to -32.5	-26.5 to -31.6	-26.5 to -31.5

**Table 4:** Summary of compound-specific δ^13^C values from EBA and LBA/EIA charred crust residues. Data is presented in Online Resource 2

	EBA (*n* = 6)		LBA/EIA (*n* = 5)	
	δ^13^C_16:0_ ‰	δ^13^C_18:0_ ‰	δ^13^C_16:0_ ‰	δ^13^C_18:0_ ‰
Mean (1 s.d.)	-30.3 ± 1.8	-30.4 ± 1.8	-23.2 ± 2.3	-22.3 ± 3.1
Range	-27.2 to -33.6	-26.6 to -33.6	-19.7 to -27.3	-19.1 to -28.0

**Fig. 6:**
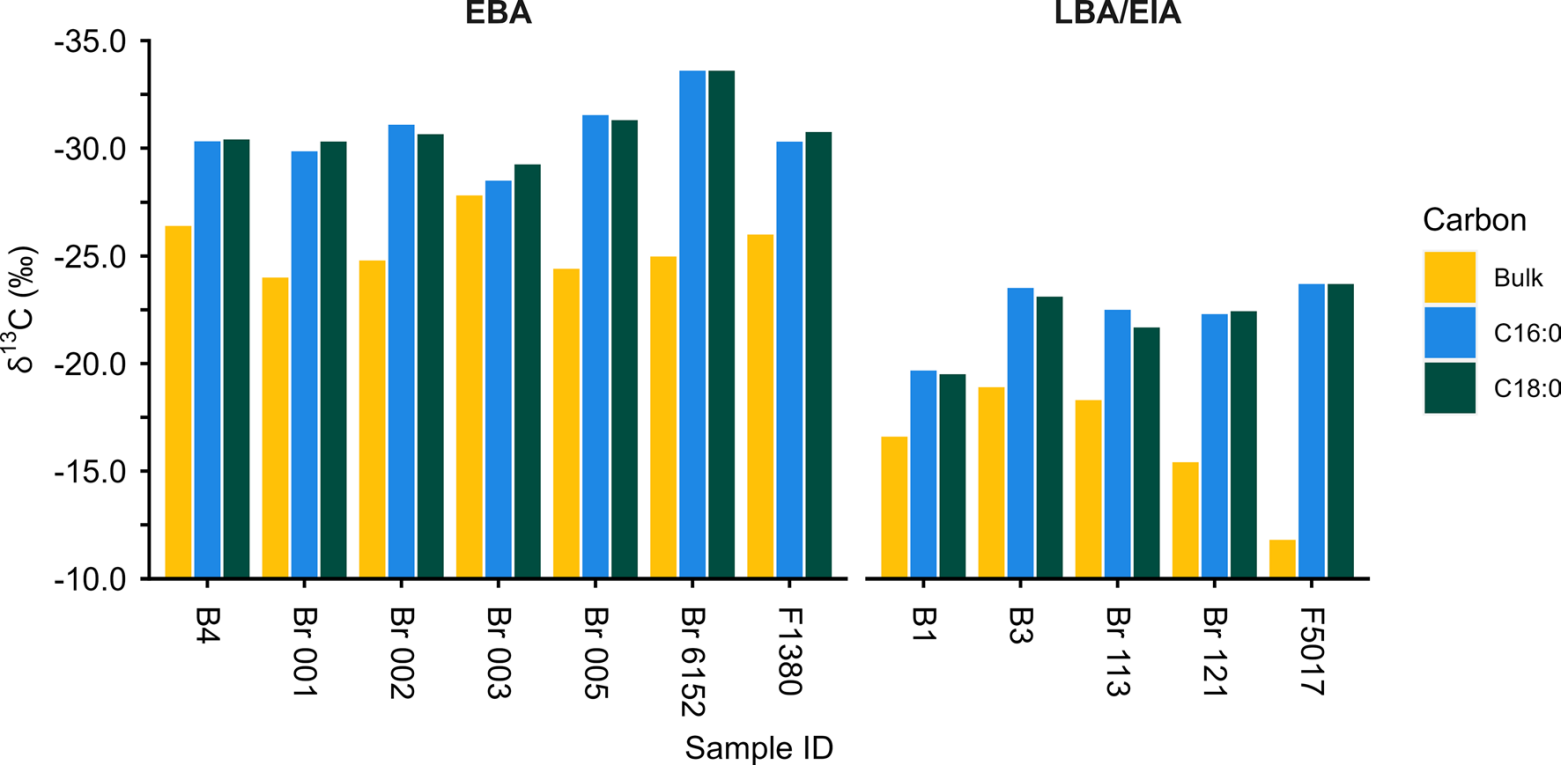
Histogram comparing the bulk δ^13^C, δ^13^C_16:0_ and δ^13^C_18:0_ values of EBA and LBA/EIA charred crusts. Data is presented in Online Resource 2

**Fig. 7:**
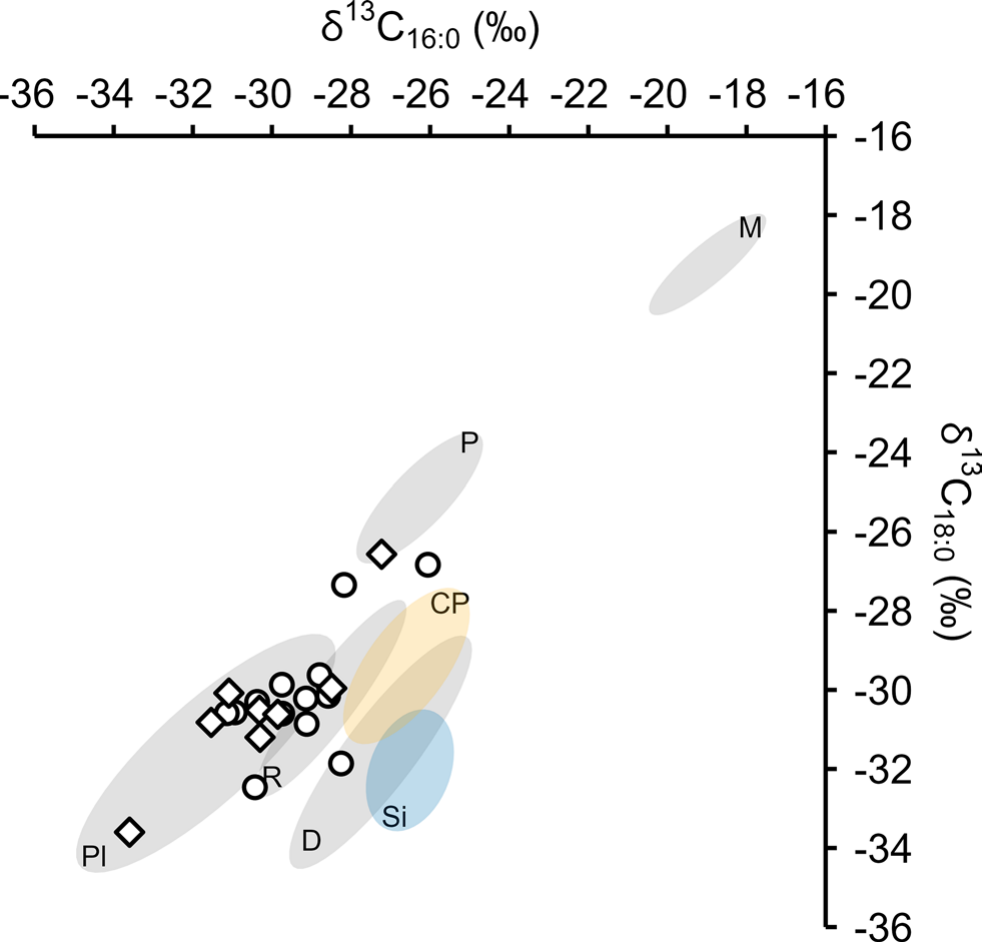
Plot of δ^13^C_16:0_ versus δ^13^C_18:0_ values of EBA samples against 1σ reference ellipses of modern carbon-corrected foodstuffs (**D** = Dairy, **M** = *P. miliaceum*, **P** = Porcine, **Pl** = C_3_ Plants, **R** = ruminant adipose) and Polish Neolithic ceramic-absorbed residues (**CP** = Cooking Pots, **Si** = Sieves, Salque et al. 2013). **Circles** = ceramics, **Diamonds** = charred crusts. Archaeological and modern data are presented, with references, in Online Resource 2

The δ^13^C_16:0_ and δ^13^C_18:0_ values of all EBA samples are within the range of C_3_ carbon sources and are depleted from bulk δ^13^C values as expected (Ballentine et al. 1998; Craig et al. 2007; Fig. 6). Most EBA samples plot in the range of C_3_ plant and ruminant adipose fats (Fig. 7), corresponding to their dominance in archaeobotanical and zooarchaeological assemblages (Kroll 2010; Müller and Kneisel 2010; Makowiecki 2015).

Three EBA residues plot either within or near the range of porcine products and appear distinct from the predominant C_3_ plant/ruminant adipose residues, although it is not possible to assess this distinction statistically due to the small sample size. This observation may indicate that porcine and ruminant products were not regularly processed in the same vessels, either concurrently or sequentially, although further investigation is necessary to demonstrate this. The low frequency of porcine residues is consistent with the zooarchaeological record.

One sample produced a Δ^13^C (δ^13^C_18:0_ - δ^13^C_16:0_) value of -3.6‰, placing it as an outlier among the EBA ceramic residues (Z-score = 3), suggesting either a wild ruminant or dairy origin (Fig. 7). The sample does not plot within the range of Polish Neolithic ceramic sieves that were reportedly used for processing dairy products (Fig. 7, Salque et al. 2013). However, this difference may be explained by either a contribution of non-dairy lipids or a difference in environmental conditions between sites (Regert 2011). The scarcity of dairy residues is consistent with limited zooarchaeological evidence for dairying at Bruszczewo (Makowiecki 2015).

One EBA charred crust, Br 6152, produced extremely low δ^13^C_16:0_ and δ^13^C_18:0_ values, within the range of C_3_ plant products (Fig. 7), in addition to a low δ^15^N value (3.8) and high P/S (4.9) and E/H (5.5) values, relative to other EBA charred crusts. This residue had a high abundance of polyunsaturated fatty acids and a suite of phytosterols, indicating that it predominantly, if not entirely, comprises C_3_ plant products. The sample was from a loose, charred accumulation formed in the bottom of a vessel, perhaps suggesting that it was the product of either different ingredients or cooking methods, compared to those forming the other charred crusts that generally adhere to higher sections of vessel walls. Further characterisation of this charred mass requires microscopic investigation to evaluate the presence of plant remains and determine whether it derived from either a bread or porridge type product (González-Carretero et al. 2017; Valamoti et al. 2019). Nonetheless, this sample indicates that a range of culinary activities were undertaken at Bruszczewo during the EBA.

**Fig. 8:**
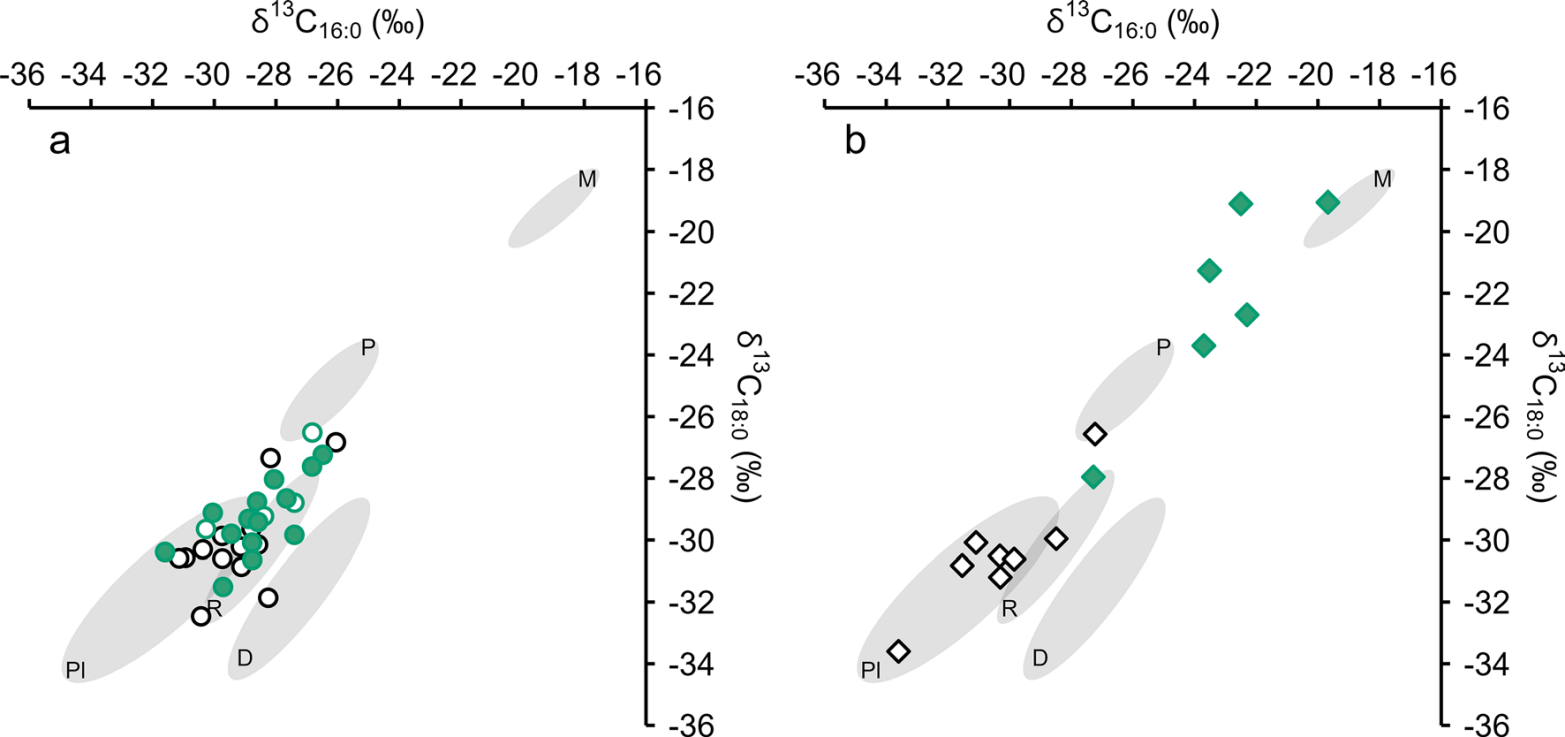
Plots of δ^13^C_16:0_ versus δ^13^C_18:0_ values of EBA (**black**) and LBA/EIA (**green**) ceramic-absorbed (**a**,** circles**) and charred crust (**b**,** diamonds**) residues against 1σ reference ellipses of modern carbon-corrected foodstuffs (**D** = Dairy, **M** = *P. miliaceum*, **P** = Porcine, **Pl** = C_3_ Plants, **R** = ruminant adipose). Filled symbols contain miliacin. Archaeological and modern data are presented, with references, in Online Resource 2

Miliacin is present in 14 LBA/EIA ceramic-absorbed residues analysed by GC-C-IRMS. However, there is no significant difference between the δ^13^C_16:0_ and δ^13^C_18:0_ values of EBA and LBA/EIA ceramic-absorbed residues indicative of a contribution of ^13^C-enriched lipids (Fig. 8). This observation is comparable to data from the Iron Age site of Vix-Mont Lassois, France, where δ^13^C_16:0_ and δ^13^C_18:0_ values of ceramic-absorbed residues are indistinguishable, regardless of the presence or absence of miliacin (Rageot et al. 2019). These results are not wholly unexpected, as miliacin generally comprises < 1% of ceramic-absorbed residues from Bruszczewo, yet they are in stark contrast to significantly ^13^C-enriched LBA/EIA charred crusts (Fig. 8). There is, however, no apparent relationship between miliacin abundance and ^13^C enrichment in either ceramic-absorbed or charred crust residues.

**Fig. 9:**
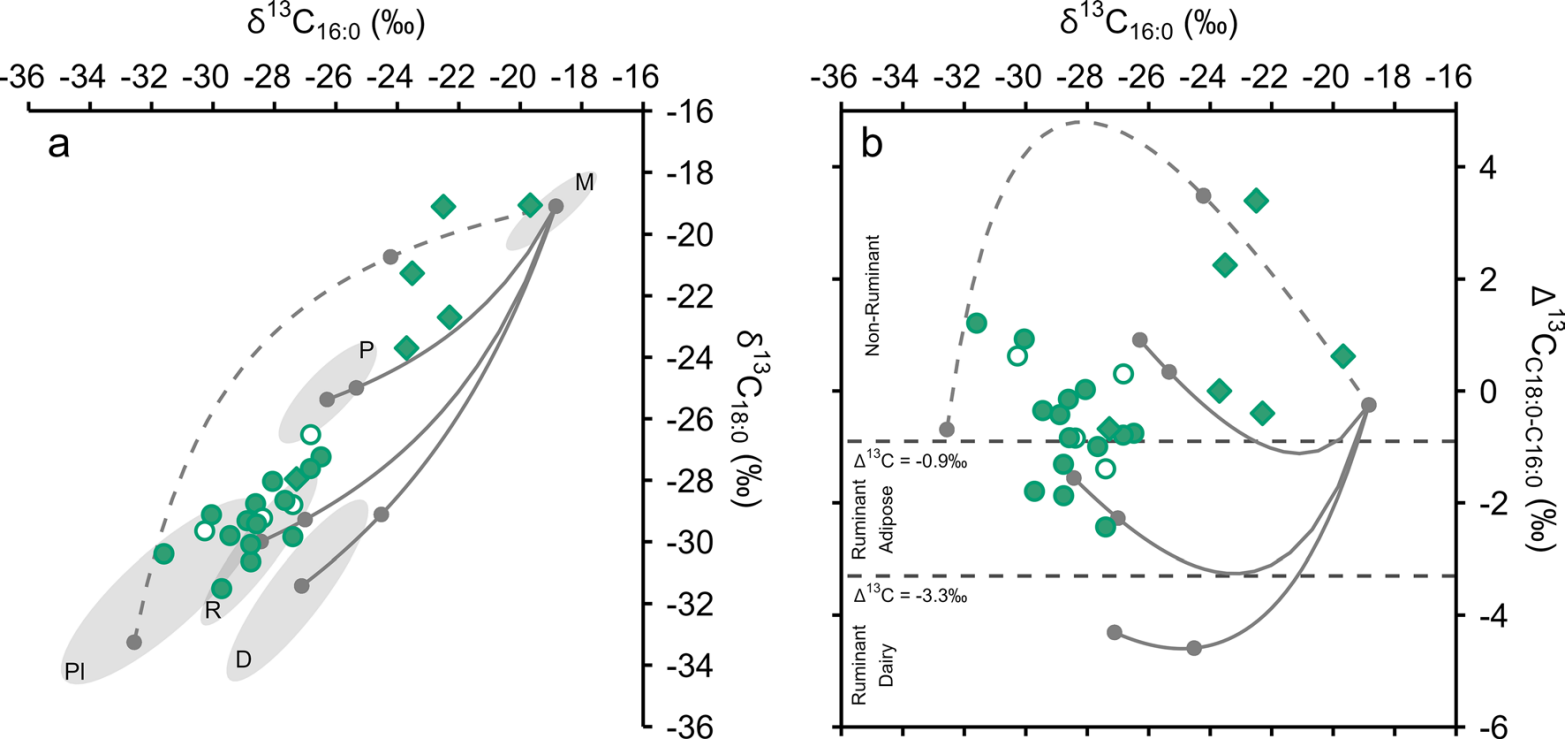
**a** – Plot of δ^13^C_16:0_ versus δ^13^C_18:0_ values of LBA/EIA samples against 1σ reference ellipses of modern carbon-corrected foodstuffs (**D** = Dairy, **M** = *P. miliaceum*, **P** = Porcine, **Pl** = C_3_ Plants, **R** = ruminant adipose). **b** – Plot of δ^13^C_16:0_ versus Δ^13^C (δ^13^C_18:0_ - δ^13^C_16:0_) values of LBA/EIA samples. Overlain are theoretical mixing lines of modern carbon-corrected foodstuffs, with points marking 0, 50, and 100% mixtures of lipids by dry weight. The mixing of C_3_ plants (dashed line) is modelled exclusively from C_3_ cereals (wheat, oat, and barley). **Circles** = ceramics, **Diamonds** = charred crusts, and filled symbols contain miliacin. Archaeological and modern data are presented, with references, in Online Resource 2

Most LBA/EIA ceramic-absorbed residues plot within the ranges of ruminant adipose and C_3_ plant lipids, with Δ^13^C values > -0.9‰ observed in many residues indicating a dominant contribution of C_3_ plant lipids (Fig. 9). Porcine and dairy products are unlikely to have been commonly processed, although their processing in mixed use vessels cannot be completely excluded. Consideration was given to the influence of ^13^C-enriched carbon, from *P. miliaceum*, on the isotopic composition of these residues (Hendy et al. 2018), as miliacin was frequently present. Theoretical δ^13^C_16:0_ and δ^13^C_18:0_ values were proportionally calculated for mixtures of different resources, taking into account their dry weight lipid content, to further define and distinguish residue origin (Fig. 9). The mixing models indicate that few LBA/EIA ceramic-absorbed residues are likely to exclusively comprise either C_3_ or C_4_ plant lipids. A dominant contribution of lipids from *P. miliaceum* is not observed in any ceramic-absorbed residue. However, mixing models incorporating animal resources demonstrate that ^13^C enrichment is barely discernible even when *P. miliaceum* comprises 50% of processed mixtures, by dry weight (Fig. 9), reflecting the low lipid content of the cereal, relative to animal resources, and the limited influence of ^13^C-enriched carbon in predominantly C_3_ isoscapes. Therefore, there is potential for the underestimation of *P. miliaceum* processing, in residues comprising animal lipids, based on isotopic composition. Experiments are necessary to demonstrate how these results relate to authentic culinary activities, particularly when more than two ingredients are combined.

Miliacin was identified in all six LBA/EIA charred crust residues analysed by GC-C-IRMS (Fig. 8). According to the dry weight mixing model (Fig. 9), it is likely that five of these residues derive from processed mixtures comprising > 50% *P. miliaceum*. These residues comprise high abundances of phytosterols (e.g. Figure 3) and produce high C/N, P/S, E/H, and Δ^13^C values that indicate a dominant contribution of plant tissue to charred crusts. Conversely, one residue is within the range of ruminant adipose lipids, with the processed mixture likely comprising < 50% *P. miliaceum*, corresponding to a lower abundance of miliacin and lower P/S, E/H, and Δ^13^C values that indicate a greater contribution of animal tissue to this charred crust (Table 5). This dataset indicates that, while *P. miliaceum* may have been a minor ingredient throughout the use-life of many vessels (long-term, ceramic-absorbed residues), the cereal was a dominant ingredient in some meals prepared at Bruszczewo (short-term, charred crust residues).

Compound-specific δ^13^C values were obtained from both ceramic-absorbed and charred crust residues from four EBA and three LBA/EIA sherds (Table 5), enabling the comparison of long- and short-term culinary activities (Miller et al. 2020). Ceramic-absorbed residues do not contain phytosterols and generally produce lower E/H and Δ^13^C values than their respective charred crusts. This indicates a greater contribution of plant lipids to charred crusts and may suggest that different meals were processed throughout vessel use-life, yet these data may also reflect biases in the representation of plant products in either ceramic-absorbed or charred crust residues. The similarity of compound-specific δ^13^C values obtained from Br 102, relative to the values obtained from other vessels, may support the former interpretation, if one assumes consistency in any biases. However, further research is necessary to assess the dynamics of residue formation and degradation between these two materials.

**Table 5:** Comparison of molecular and isotopic data obtained from ceramic and charred crust samples from the same sherds. EBA = br 0## and LBA/EIA = br 1##. For sterols, **C** = cholesterol and its derivatives, **P** = phytosterols (Sitosterol, Stigmasterol, campesterol) and their derivatives

ID	Miliacin		Sterols		E/H		Compound-Specific (‰)						Bulk (‰)	
							Ceramic			Crust			Crust	
	Ceramic	Crust	Ceramic	Crust	Ceramic	Crust	C_16:0_	C_18:0_	Δ^13^C	C_16:0_	C_18:0_	Δ^13^C	δ^13^C	δ^15^N
Br 001	-	-	C	C P	3.9	4.5	-29.1	-30.9	-1.7	-29.9	-30.6	-0.8	-24.0	5.2
Br 003	-	-	C	C	1.9	2.7	-29.1	-30.2	-1.1	-28.5	-30.0	-1.5	-27.8	6.4
Br 005	-	-	-	C P	3.7	5.3	-28.8	-29.6	-0.8	-31.5	-30.8	0.7	-24.4	4.5
Br 017	-	-	C	-	2.8	3.1	-26.1	-26.8	-0.8	-27.2	-26.6	0.7	-	-
Br 102	X	X	C	C P	-	1.0	-26.5	-27.2	-0.8	-27.3	-28.0	-0.7	-	-
Br 113	X	X	-	P	5.5	5.1	-28.1	-28.0	0.0	-22.5	-19.1	3.4	-18.3	0.9
Br 121	X	X	C	C P	1.1	4.0	-28.6	-29.4	-0.8	-22.3	-22.7	-0.4	-15.4	1.2

### Addressing the research questions

*Is the apparent absence of P. miliaceum in the EBA supported by the analysis of a larger and more diverse sample set?*

There is no evidence of *P. miliaceum* processing among EBA samples. These results correspond to recently published radiocarbon dates suggesting that *P. miliaceum* was introduced into the region after the end of EBA occupation at Bruszczewo ca. 1500 BC (Czebreszuk et al. 2015; Filipović et al. 2020). Individual *P. miliaceum* caryopses observed in EBA contexts at Bruszczewo are most probably intrusive from later periods. Furthermore, sherds with millet impressions, recovered from the transgression layer between EBA and LBA/EIA contexts, can likely be attributed to the later period of occupation.

*How prevalent was P. miliaceum processing in the LBA/EIA?*

Through the detection of miliacin, in either ceramic-absorbed or charred crust residues, a minimum of 19 of the 39 LBA/EIA vessels sampled are confirmed to have been used to process *P. miliaceum* during their use-life. An additional 10 vessels with ^13^C-enriched (bulk) charred crusts also strongly suggest *P. miliaceum* processing, given that there is no evidence that other ^13^C enriched products were processed at Bruszczewo. While at least half of the vessels sampled were used to process *P. miliaceum* at some point during their use-life, it is not easy to establish the frequency with which the cereal was used. The archaeobotanical record indicates that *P. miliaceum* was a minor cereal, relative to barley, although certain biases may underrepresent its true scale of exploitation (Märkle and Rösch 2008; Motuzaitė Matuzevičiūtė et al. 2012). Conversely, the ORA dataset may be subject to as yet unidentified biases influencing the transfer, persistence and preservation of miliacin, relative to other compounds, as has been examined for other cereals (Hammann and Cramp 2018). For example, it is possible that either the cooking of *P. miliaceum*, or meals into which it was incorporated, may have been more likely to form charred crusts, thereby overrepresenting their frequency in this dataset. Finally, one must consider the role of aceramic processing methods, in either the complete or partial production of meals, and their influence on the representation of certain ingredients. A greater understanding of the extent to which *P. miliaceum* was consumed at Bruszczewo may be achieved via isotopic analysis of human and animal remains. Additionally, employing solvent extraction in future ORA investigations may identify the presence of alkylresorcinols and help investigate the use of other cereals (Colonese et al. 2017).

If it is presumed that biases in the representation of *P. miliaceum* processing in ceramic-absorbed residues are comparable between contemporary sites, it appears that *P. miliaceum* processing was more prevalent at Bruszczewo than the contemporary funerary site of Maciejowice, Poland, where only 4 of 12 ceramic-absorbed residues analysed contained miliacin (Isaksson and Nilsson 2018). This may represent the processing of *P. miliaceum* in either greater quantities or a wider range of contexts. Regional archaeobotanical datasets indicate varied degrees of *P. miliaceum* exploitation during the LBA/EIA (Moskal-del Hoyo et al. 2015; Kapcia and Mueller-Bieniek 2019; Mueller-Bieniek et al. 2019), yet further research is necessary to understand and explain these differences at contemporary sites with similar functions, such as Bruszczewo and Maciejowice, where funerary and ceremonial events appear to have taken place. Evidence for the consumption of the cereal at Bruszczewo appears to be in stark contrast to the apparent absence of human isotopic evidence of millet consumption in Central Poland during the Bronze Age (Pospieszny et al. 2021). However, potential biases in isotopic datasets (Lee-Thorp 2008; Pospieszny et al. 2021), the absence of comparable isotopic data from Bruszczewo, and ORA data from elsewhere, presently precludes a fuller consideration of this issue.

*How was P. miliaceum prepared, consumed, and incorporated into culinary culture?*

The results indicate that *P. miliaceum* was incorporated into a variety of culinary activities, including concurrent and sequential mixing, primarily with C_3_ plant and ruminant meat products, in multi-use vessels. The use of *P. miliaceum* as a minor and major ingredient in different meals, and its absence in others, perhaps indicates its role as a staple product employed in either a diverse subsistence strategy or varied set of culinary activities. Indeed, the diversity of Lusatian Culture ceramic types and forms from Bruszczewo allude to varied and distinct processes of food production and consumption. However, the significance of *P. miliaceum* is somewhat dependent upon the scale of its exploitation and function of the site itself. There is no evidence to suggest that *P. miliaceum* processing was associated with either specific vessel types or decorative features, although diagnostic information for the sherds sampled was limited. Further investigation of sherds with more clearly defined typological and decorative characteristics is desirable. Miliacin was identified in the sherd with millet impressions, although this is most likely from processing *P. miliaceum*, as compounds present in clays and organic tempers are unlikely to survive the firing process (Reber et al. 2019). Evidence for *P. miliaceum* processing was observed in residues from each of the three trenches examined in this study, yet there is no evidence to suggest that culinary activities differed between either trenches or stratigraphic units.

## Conclusions

This study has developed upon preliminary research undertaken at Bruszczewo (Heron et al. 2016) by applying EA-IRMS, GC-MS, and GC-C-IRMS to a greater number of charred crusts (*n* = 20), in addition to applying GC-MS and GC-C-IRMS to a large number of ceramic-absorbed residues (*n* = 42). This analysis demonstrates that at least half of the LBA/EIA vessels investigated were used to process *P. miliaceum*, in a variety of culinary activities. Although the sample size is small, there is no evidence to suggest that *P. miliaceum* was processed in ceramic vessels during the EBA. The multifunctional role of ceramics has been revealed, alongside the processing of other plant and animal resources, highlighting the complexity of culinary activities practiced at Bruszczewo during prehistory.

## Electronic supplementary material

Below is the link to the electronic supplementary material.

Supplementary Material 1

Supplementary Material 2

## Data Availability

No datasets were generated or analysed during the current study.
