# Supplementary material for: Millet and meals: the role and significance of Panicum miliaceum in culinary contexts at Bruszczewo, Poland
Source: Archaeol Anthropol Sci. 2024 Dec 4;17(1):1. doi: 10.1007/s12520-024-02095-1 (PMC11615104; doi:10.1007/s12520-024-02095-1)
Supplement: Supplementary file 1 — Supplementary Material 1 [file 12520_2024_2095_MOESM1_ESM.docx]

**Online Resource 1**

**Millet and Meals: The role and significance of *Panicum miliaceum* in culinary contexts at Bruszczewo, Poland**

**Archaeological and Anthropological Sciences**

**Authors:**

Edward A. Standall^a,b,*^, Oliver E. Craig^b^, Jutta Kneisel^c^, Johannes Müller^c^, Wiebke Kirleis^c^, Janusz Czebreszuk^d^ and Carl Heron^a^

**Affiliations:**

^a^ Department of Scientific Research, British Museum, London, United Kingdom, WC1B 3DG

^b^ BioArCh, Department of Archaeology, University of York, York, United Kingdom, YO10 5DD

^c^ Institute of Pre- and Protohistory, Kiel University (Christian-Albrechts-Universität zu Kiel), Kiel, Germany, 24118

^d^ Faculty of Archaeology, Adam Mickiewicz University, Poznań, Poland, 61-614

^*^ Nara National Research Institute for Cultural Properties, Nara, Japan, 630-8577

**Corresponding Author:**

Edward A. Standall [e.standall@outlook.com](mailto:e.standall@outlook.com)

**ORCID:**

Standall, E.A. - 0000-0003-1342-0728

Craig, O.E. - 0000-0002-4296-8402

Kneisel, J. - 0000-0001-7893-790X

Müller, J. - 0000-0002-3538-197X

Kirleis, W. - 0000-0003-0229-8958

Czebreszuk, J. - 0000-0001-8487-6031

Heron, C. - 0000-0002-5206-7464

**Methods:**

A clean scalpel was used to remove charred crusts from ceramics, with material stored in aluminium foil packets that were refrigerated within days of collection. Charred crusts were not subjected to pre-treatment, in-line with the methodology previously employed by Heron *et al*. (2016). Bulk δ^13^C and δ^15^N values were acquired for samples of around 1 mg, measured in duplicate, using a Sercon (Crewe, UK) continuous flow 20-22 Isotope Ratio Mass Spectrometer with universal Faraday triple collectors (C, N, S, O) and additional single Faraday collector for m/z 3 (H). This was coupled to an EA-GSL preparation unit with a 66 place open carousel autosampler. Uncertainties of each measurement were calculated according to Kragten (1994), combining uncertainty values of international standards and repeat measurements of samples and reference materials. Data was corrected with international standards, IAEA 600 caffeine, IAEA N2 ammonium sulphate, and IA R006 cane sugar (IsoAnalytical). Results were reported in per mil (‰) relative to standards. The precision of charred crust samples from Bruszczewo was 0.05 (1σ) for δ^13^C and 0.18 (1σ) for δ^15^N.

Ceramic samples were obtained in a powdered form using a rotary drill. The sampling area was cleaned, using the drill, by removing the top surface layer of the sherd. A clean drill bit was used for taking the sample. Samples of archaeological sherds were obtained from an approximately 2 x 2 cm area on the internal surface, away from the sherd edge, up to a depth of 0.5 cm.

Simultaneous extraction and derivatisation of lipids was achieved using an adapted acid methanol methodology (Correa-Ascencio and Evershed 2014). Methanol was added to homogenised samples of ceramic powders (4 mL to 1 g), and charred crusts (1 mL to 20 mg). Mixtures were ultrasonicated for 15 minutes, before adding concentrated (98%) sulphuric acid (H_2_SO_4_), and heating, at 70°C, for four hours. The volume of H_2_SO_4_ used was proportional to the volume of methanol, with 800 μL used for ceramic powder and 200 μL for charred crust samples. Following centrifugation, at 3000 rpm for five minutes, extracts were transferred to a separate vial. Lipids were extracted from the acidified mixture using n-hexane (3 x 2 mL) and dried under a gentle stream of nitrogen. Samples were resuspended in 100 μL of n-hexane and transferred to a conical insert, in a GC vial, with 10 μL of a C36 alkane standard that was agitated, before analysis, to ensure homogenisation. A C_16:0_ and C_18:0_ fatty acid standard was processed with each batch of around 10 samples, in addition to a method blank.

GC-MS analysis was undertaken using an Agilent 7890A Series Gas Chromatograph coupled to an Agilent 5977B Mass-selective detector equipped with a quadrupole mass analyser (Agilent technologies, Cheadle, Cheshire, UK). A split/splitless injector, operated in splitless mode with a purge flow of 50 mL min^-1^, was maintained at 300°C and a DB-5MS column was used (30 m x 250 μm x 0.25 μm, Agilent technologies, Cheadle, Cheshire, UK). Helium was the carrier gas (2 mL min^-1^). The oven temperature was set to 50°C for 2 minutes, then increased at 10°C min^-1^ to 200°C before increasing at 4°C min^-1^ to 320°C and held for 5 minutes. The ionization energy of the mass spectrometer was 70 eV and spectra were obtained in scanning mode between m/z 50 and 800. The transfer line temperature was held at 280°C.

Selective ion monitoring (SIM) was undertaken using an Agilent 7890A Series Gas Chromatograph coupled to an Agilent 5977B Mass-selective detector equipped with a quadrupole mass analyser (Agilent Technologies, Cheadle, Cheshire, UK). A split/splitless injector, operated in splitless mode with a purge flow of 50 mL min-1, was maintained at 300°C and a (50%-cyanopropyl)-methylpolysiloxane DB-23 column was used (PN 122-2362; 60 m x 250 μm x 0.25 μm; J&W Scientific technologies, Folsom, CA, USA). Helium was the carrier gas (1.5 mL min^-1^). The oven was set to 50°C for 2 minutes, increased at 10°C min^-1^ to 100°C, increased at 4°C min^-1^ to 140°C, increased at 0.5°C min^-1^ to 160°C, then increased at 20°C min^-1^ to 250°C and held for 10 minutes. SIM mode was used in order to target the specific markers of aquatic resources using characteristic ion groups: m/z 74, 87, 213, 270 for 4,8,12-trimethyltridecanoic acid (4,8,12-TMTD), m/z 74, 88, 101, 312 for pristanic acid, m/z 74, 101, 171, 326 for phytanic acid, and m/z 74, 105, 262, 290, 318, 346 for the detection of ω-(o-alkylphenyl)alkanoic acids of carbon lengths C_16_ to C_22_ (APAA_16-22_). The transfer line temperature was held at 280°C.

MassHunter software (Agilent Technologies) and the NIST 17 mass spectra database was used for quantitative and qualitative analysis of extracts.

Compound-specific carbon isotope (GC-C-IRMS) analysis of methyl palmitate and methyl stearate was undertaken using an Isoprime 100 (Isoprime, Cheadle, UK) IRMS detector coupled to an Agilent 7890B Series Gas Chromatograph with an Isoprime GC5 interface. An 5975C inert XL mass-selective detector (MSD) equipped with a quadrupole mass analyser was also coupled to the GC. A split/splitless injector, operated in splitless mode with a purge flow of 50 mL min^-1^, was maintained at 300°C and a DB-5MS UI column was used (PN 122-5562UI; 60 m x 250 μm x 0.25 μm; J&W Scientific technologies, Folsom, CA, USA). Helium (ultra-high purity grade) was the carrier gas (2 mL min^-1^). The oven temperature was set to 50°C for 0.5 minutes, increased at 25°C min^-1^ to 175°C, then increased at 8°C min^-1^ to 325°C and held for 20 minutes. Gases eluted from the column were split into two streams, that entered the MSD and GC5 interface. The ionization energy of the mass spectrometer was 70 eV and spectra were obtained in scanning mode between m/z 50 and 800. The GC5 furnace tube (CuO) was maintained at 850°C to oxidise carbon species to CO_2_. Clear resolution and baseline separation of analysed peaks was achieved. Products eluted from the furnace were ionised in the IRMS by electron impact. Ion intensities of m/z 44, 45 and 46 were recorded for automatic computing of the ^13^C/^12^C ratio of each peak in the extracts.

Computation was made with IonVantage and IonOS softwares (Isoprime, Cheadle, UK). The results of the analysis were expressed in per mill (‰) relative to an international standard (V-PDB). The accuracy and precision of the instrument was determined on n-alkanoic acid ester standards of known isotopic composition (Indiana standard F8-3). The mean ± S.D. value was -29.96 ± 0.11‰ for the methyl ester of C_16:0_ (reported mean value vs. VPDB -29.90 ± 0.03‰) and -23.31 ± 0.08‰ for C_18:0_ (reported mean value vs. VPDB -23.24 ± 0.01‰). Each sample was measured in duplicate with a mean of S.D. 0.30‰ C_16:0_ and 0.26‰ for C_18:0_. Values were also corrected after analysis to account for the methylation of the carboxyl group that occurs during acid extraction. Corrections were based on comparisons with a standard mixture of C_16:0_ and C_18:0_ fatty acids of known isotopic composition processed in each batch under identical conditions. The results from the analysis are reported in parts per mil (‰) relative to an international standard (V-PDB). Each batch of samples was calibrated using a calibration curve (average R2 = 0.998) in 1 batch, based on expected vs. measured δ^13^C values of n-alkanes and n-alkanoic acid esters international standards (Indiana A6 and F8-3 mixture). More precisely, the accuracy of the instrument was determined on n-alkanoic acid ester standards of known isotopic composition (Indiana standard F8-3, 4 measurements).
